# Coronary artery lumen complexity as a new marker for refractory symptoms in patients with vasospastic angina

**DOI:** 10.1038/s41598-020-79669-1

**Published:** 2021-01-08

**Authors:** Atsushi Tanaka, Akira Taruya, Kyosuke Shibata, Kota Fuse, Yosuke Katayama, Mao Yokoyama, Manabu Kashiwagi, Ota Shingo, Takashi Akasaka, Nobuhiro Kato

**Affiliations:** 1 Department of Cardiovascular Medicine, Wakayama Medical University, 811-1 Kimiidera, Wakayama, 641-8510 Japan; 2 Faculty of Biology-Oriented Science and Technology, Kindai University, 930 Nishimitani, Kinokawa, Wakayama 649-6493 Japan

**Keywords:** Cardiology, Cardiovascular biology, Interventional cardiology, Applied optics

## Abstract

Refractory angina is an independent predictor of adverse events in patients with vasospastic angina (VSA). The aim of this study was to investigate the relationship between coronary lumen complexity and refractory symptoms in patients with VSA. Seventeen patients with VSA underwent optical coherence tomography. The patients were divided into the refractory VSA group (n = 9) and the stable VSA group (n = 8). A shoreline development index was used to assess the coronary artery lumen complexity. Shear stress was estimated using a computational fluid dynamics model. No difference was observed in the baseline characteristics between the two groups. The refractory VSA group showed the higher shoreline development index (refractory VSA 1.042 [1.017–1.188] vs stable VSA 1.003 [1.006–1.025], p = 0.036), and higher maximum medial thickness (refractory VSA 184 ± 17 μm vs stable VSA 148 ± 31 μm, p = 0.017), and higher maximum shear stress (refractory VSA 14.5 [12.1–18.8] Pa vs stable VSA 5.6 [3.0–10.5] Pa, p = 0.003). The shoreline development index positively correlates with shear stress (R^2^ = 0.46, P = 0.004). Increased medial thickness of the coronary arteries provokes lumen complexity and high shear stress, which might cause refractory symptoms in patients with VSA. The shoreline index could serve as a marker for irritability of the medial layer of coronary arteries and symptoms.

## Introduction

Vasospastic angina (VSA) is an important clinical disorder primarily attributable to coronary artery spasm^1^. VSA was first reported in 1959 as a variant angina, characterized by nitrate-responsive resting angina with transient ST segment elevation^2^. Variant angina is now considered as a specific form of VSA. However, Prinzmetal and his colleagues' careful clinical observations are still important in our practice, as clinical manifestations of resting angina alone have been established as independent risk factors for poor prognosis in patients with VSA^3^. Vasodilators have been used for the treatment of VSA to prevent and relieve symptoms of angina^4^. However, clinicians sometimes face challenges with treatment strategies for patients of VSA who manifest refractory symptoms despite optimal medication^4,5^. Furthermore, frequent angina is one of the independent predictors for adverse events in patients with VSA receiving vasodilator therapy^6^. Symptoms are an important consideration in the treatment of VSA^3^, and there is scarce data to explain the reasons for refractory symptoms in some patients with VSA.

In normal vascular homeostasis, coronary flow induces dose-dependent secretion of vasodilators, including nitric oxide (NO) and prostacyclin^7^. These lead to relaxation of the smooth muscles cells in the medial layer of coronary arteries and decrease the vascular tone to maintain constant shear stress^8^. However, in recent times, coronary lumen complexity has been found in patients with VSA, even during the asymptomatic stage^9^. We hypothesized that VSA patients with refractory symptoms show more abnormal responses in their shear stress compared to those with controlled VSA, resulting in further lumen complexity. The aim of this study was to investigate the relationship between coronary lumen complexity, shear stress on the coronary vascular intima, and refractory symptoms in patients with VSA using optical coherence tomography (OCT) and computational fluid dynamics (CFD).

## Material and methods

The study protocol was approved by the Ethics Committee of Wakayama Medical University.

This study complies with the Declaration of Helsinki. Informed consent was obtained from all participants.

### Subjects

We included 23 patients with VSA who underwent OCT before intracoronary administration of nitroglycerin. VSA was diagnosed on the basis of criteria mentioned in the Guidelines for Diagnosis and Treatment of Patients with Vasospastic Angina, by the Japanese Circulation Society^4^. It was the first provocation test in 12 (71%) of 17 symptomatic patients who showed ischemic ST-T changes in ECG or Holter ECG. For the remaining five patients who had positive results in the previous provocation test, we performed coronary angiography and a provocation test due to the development of exercise-induced angina or different type of angina. The responsible coronary artery was determined by a combination of ECG findings at the time of acute angina or from the results of previous provocation tests. All medications were continued until coronary angiography was performed. In our previous study, we reported that patients with VSA showed lumen irregularity even at asymptomatic status. This lumen irregularity completely disappeared after the administration of intracoronary nitrates^9^, thus, in this study, OCT was performed for the coronary artery responsible for the spasm before nitroglycerin was administered. Patients who had more than 50% stenosis after intracoronary administration of nitroglycerin as seen on quantitative coronary angiography (n = 3), those in whom the site of spasm showed a side branch (n = 1), or those with inadequate OCT images (n = 2) were excluded from the study. In all, 17 patients were analyzed. In this study, refractory symptoms were defined as more than one event of angina per week in the nearest 4 weeks, based on a previous report^6^.

Based on the presence or absence of refractory symptoms, the patients were divided into two groups: a refractory VSA group (n = 9) and a stable VSA group (n = 8).

### OCT imaging and provocation protocol

Following a diagnostic coronary angiography without administration of nitroglycerin, pressure measurements were performed using a pressure wire (PressureWire, PressureWire, Abbott, USA). Aortic pressure at the tip of the guiding catheter and at the tip of the pressure wire was equalized. The pressure wire was then advanced to the distal part of the coronary artery. Pressure at the distal site and mean aortic pressure were simultaneously recorded, followed by OCT imaging. A frequency domain OCT catheter (ILUMIEN, Abbot Vascular, Santa Clara, California, USA, or FastView, Terumo, Tokyo, Japan) was advanced distally to the lesion. An X-ray contrast medium (Omnipaque 350 Injection, Daiichi Sankyo Co, Ltd, Tokyo, Japan) was infused through the guiding catheter at 2–4 mL/s for approximately 3–6 s using an injector pump (Mark V; Medrad, Pennsylvania, USA), followed by pullback of the OCT imaging probe at 10–40 mm/s to obtain the image of the coronary artery. To confirm the precise site of the spasm, the provocation test was performed with incremental doses of acetylcholine or ergometrine, as recommended in the guideline^4^. After administration of nitroglycerin, OCT imaging was repeated. All OCT images were digitally stored and analyzed using Image J (National Institute of Health, Bethesda, MD, USA).

### OCT image analysis

All OCT images were analyzed by 2 independent investigators (A. Taruya and Y. Katayama), who were blinded to the clinical presentations. The site showing maximum stenosis in the provocation test was selected for analysis. The most stenotic site seen on coronary angiogram during the provocation test, and the corresponding OCT image were matched using luminal configuration and anatomical landmarks such as side branches, and distance from the side branches or from the distal end. The formula for lumen area stenosis on OCT is as follows: Lumen area stenosis = (Reference lumen cross sectional area − minimum lumen cross sectional area)/reference lumen cross sectional area. An intimal bump was defined as one or more smooth projections of the intima into the lumen, according to our criteria^9^.

We used the shoreline development index to assess the complexity of the lumen circumference. The shoreline development index is used in limnology and cartography to assess the complexity of the lake shorelines^10^. The shoreline development index of the complete circle is 1. The formula for calculating the shoreline development index is as follows:

$${\text{Shoreline development index }} = {\text{ perimeter}}/\left( {{2}\surd {\text{lumen area*}}\pi } \right).$$

### Estimation of shear stress

All digitalized OCT data were transferred to a computer-aided design software (Pro/Engineer Wild Fire, Parametric Technology Corporation, Boston, MA, USA) to reconstruct three-dimensional (3D) models of the coronary arteries. The lumen contour behind the guide wire shadow was manually interpolated. The 3D volume data of 2.5-mm segments proximal and distal to the culprit lesion were then imported into COMSOL Multiphysics 4.4 software (COMSOL, Inc. Burlington, MA, USA) and was divided into small elements for analysis. The typical size of the finite element mesh used was 0.0005 mm^3^ (around 1,000,000 elements). The Navier–Stokes equations were used to determine the fluid shear stress on the luminal surface. Computations of the flow fields in all models were performed using the CFD module of the commercial finite element analysis code in COMSOL Multiphysics 4.4. Blood was assumed to be an incompressible Newtonian fluid with a density of 1060 kg/m^3^ and coefficient of viscosity of 0.005 Pa S. The difference in mean arterial pressure between the inlet and outlet of the spasm site was calculated by the subtraction of the distal site pressure from the mean aortic pressure, corrected for length. When no difference was observed in mean arterial pressure between the inlet and outlet of the spasm site, we used 0.5 mmHg for the difference in mean arterial pressure. In all simulations, flow into the artery was assumed to be pulsatile flow as in the human coronary artery.

### Statistical analysis

Statistical analysis was performed using JMP pro version 14 for Mac (SAS institute, Cary, NC, USA). Results are expressed as mean value ± standard deviation (SD) for approximately normally distributed variables and the Student’s t-test was applied for comparisons. Skewed variables are presented as medians [interquartile range] and the Wilcoxon test was used for non-parametric comparisons. Categorical variables are presented as numbers (%). The chi squared test were applied for the categorical variables. If there was an expected cell value of < 5, Fisher’s exact test was applied. The relationship between the shoreline development index and the shear stress was assessed using linear regression analysis. A p value < 0.05 was considered statistically significant.

## Results

**Figure 1:**
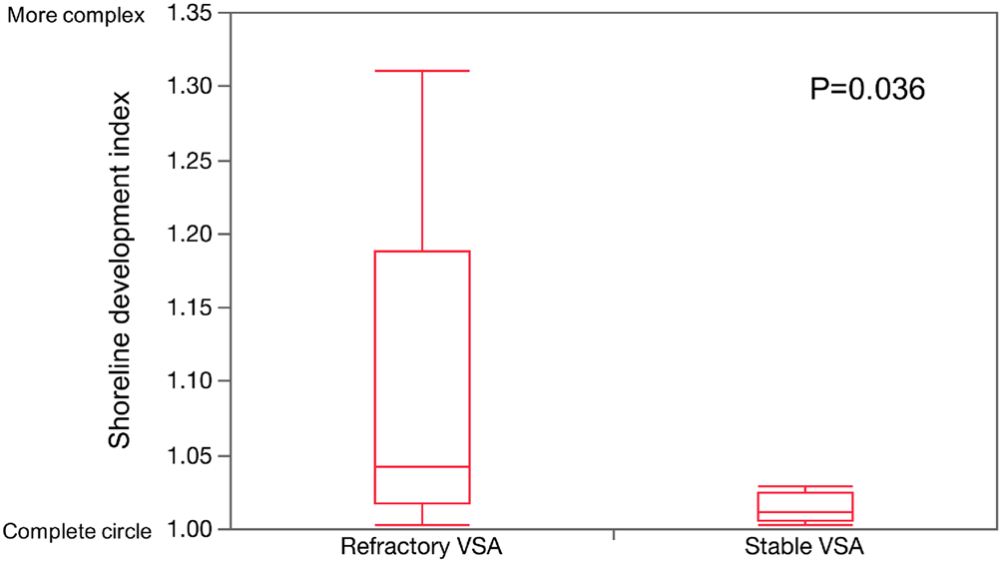
Shoreline development index. A shoreline development index is used in limnology and cartography to assess the complexity of the lake shoreline. The shoreline development index of a complete circle is 1. The shoreline development index of the refractory VSA group is larger than that of the stable VSA group (refractory VSA 1.042 [1.017–1.188] vs stable VSA 1.003 [1.006–1.025], p = 0.036).

Baseline characteristics of the patients are summarized in Table 1. No statistical difference was observed in any of the baseline characteristics between the groups. The median follow-up period from diagnosis to the provocation test was 5 (range 4–6.5) weeks. No difference was found in the duratoin of medical follow-up between the two groups (refractory VSA 5 [4.5–8] weeks vs. stable VSA 4.5^4–6^ weeks, p = 0.32). OCT findings are shown in Table 2. The distribution of the target artery was similar between the groups. The intimal bump occurred numerically more often in the refractory group, but the difference was not statistically significant (P-value of 0.06). The maximum medial thickness in the refractory VSA group was higher than that in the stable VSA group (refractory VSA group [184 ± 17 μm] vs stable VSA group [148 ± 31 μm, p = 0.017]). Figure 1 shows the shoreline development index for both groups.

**Table 1:** Patients characteristics.

	Refractory VSA (n = 9)	Stable VSA (n = 8)	*p*-value
Age, years	61.6 ± 9.6	64.3 ± 7.9	0.73
Male	7 (78)	5 (63)	0.62
Heart rate (beats per minute)	68.0 ± 7.7	70.3 ± 6.5	0.53
Systolic blood pressure (mmHg)	126.1 ± 20.0	124.9 ± 19.2	0.90
Hypertension	5 (56)	4 (50)	0.99
Dyslipidemia	3 (33)	2 (25)	0.99
Diabetes mellitus	2 (22)	2 (25)	0.99
Current smoking	3 (33)	3 (38)	0.99
Calcium channel blocker	7 (78)	4 (50)	0.33
Angiotensin II Receptor Blocker	1 (11)	3 (38)	0.29
β blocker	3 (33)	1 (13)	0.58
Nitroglycerin	1 (11)	0 (0)	0.99
Nicorandil	4 (44)	2 (25)	0.61
Reasons for provocation test			0.99
Positive ischemic findings in ECG or Holter ECG	6 (67)	6 (75)	
Development of exercise-induced angina or different type of resting angina	3 (33)	2 (25)	

Values are presented as n (%) or mean ± standard deviation.

*VSA* vasospastic angina.

**Table 2:** Optical coherence tomography findings.

	Refractory VSA (n = 9)	Stable VSA (n = 8)	*p*-value
**Target vessel**			0.87
LAD	6 (67)	6 (75)	
LCx	1 (11)	1 (13)	
RCA	2 (22)	1 (13)	
Intimal bump	7 (78)	2 (25)	0.06
Minimal lumen area (mm^2^)	5.29 ± 1.38	5.75 ± 1.16	0.73
Reference lumen area (mm^2^)	6.43 ± 1.30	7.13 ± 10.85	0.86
Lumen are stenosis (%)	81.7 ± 8.2	80.5 ± 11.1	0.41
Maximum medial thickness (μm)	184 ± 17	148 ± 31	0.017

Values are presented as n (%) or mean ± standard deviation.

*VSA* vasospastic angina, *LAD* left anterior descending artery, *LCx* left circumflex artery, *RCA* right coronary artery.

**Figure 2:**
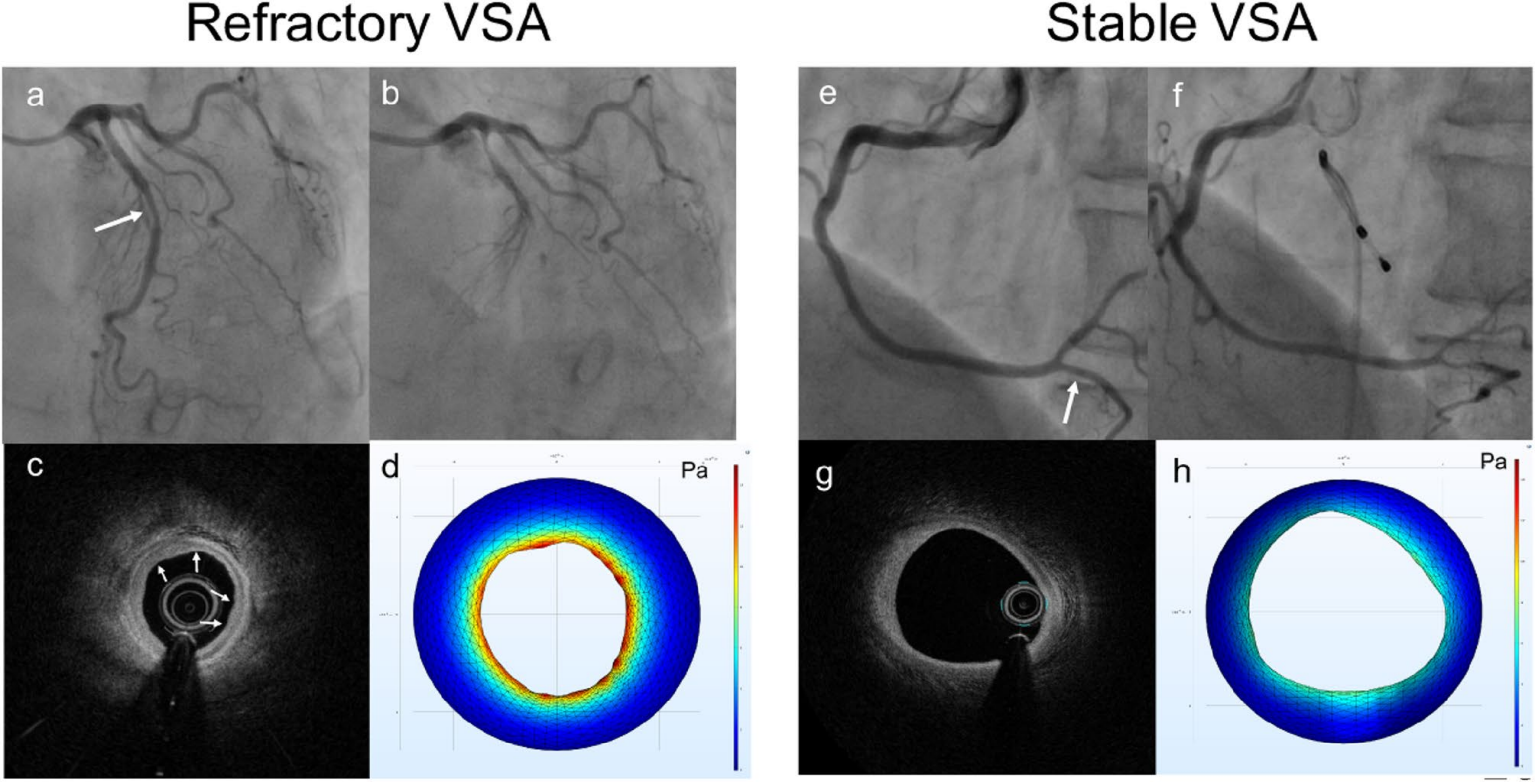
Coronary angiograms, optical coherence tomography (OCT) images, and shear stress map. (**a**,**b**) Angiograms of baseline and provocation test in the refractory vasospastic angina (VSA) group. Mild stenosis can be seen in the middle portion of the left descending coronary artery (white arrow). During the provocation test, total occlusion was observed at the mid-position of the left descending coronary artery. (**c**) OCT image at baseline in the refractory VSA group intimal bumps (white arrows) can be seen in the OCT image of the refractory VSA. The shear stress is higher in refractory VSA than in stable VSA. (**d**) Shear stress map. The shear stress is higher in refractory VSA than in stable VSA. (**e**,**f**) Angiograms of baseline and provocation tests in stable VSA. No stenosis can be seen in the angiogram at baseline (white arrow). During the provocation test, severe stenosis can be seen in the distal portion of the right coronary artery. (**g**) OCT image at baseline in stable VSA. No intimal bumps can be seen in the OCT image of the stable VSA. (**h**) Shear stress map. The shear stress has lower values in stable VSA than in refractory VSA.

The shoreline development index of the refractory VSA group was higher than that of the stable VSA group (refractory VSA group with 1.042 [1.017–1.188] vs stable VSA group with 1.003 [1.006–1.025], p = 0.036). Figure 2 presents the representative images of OCT and shear stress maps from both groups.

**Figure 3:**
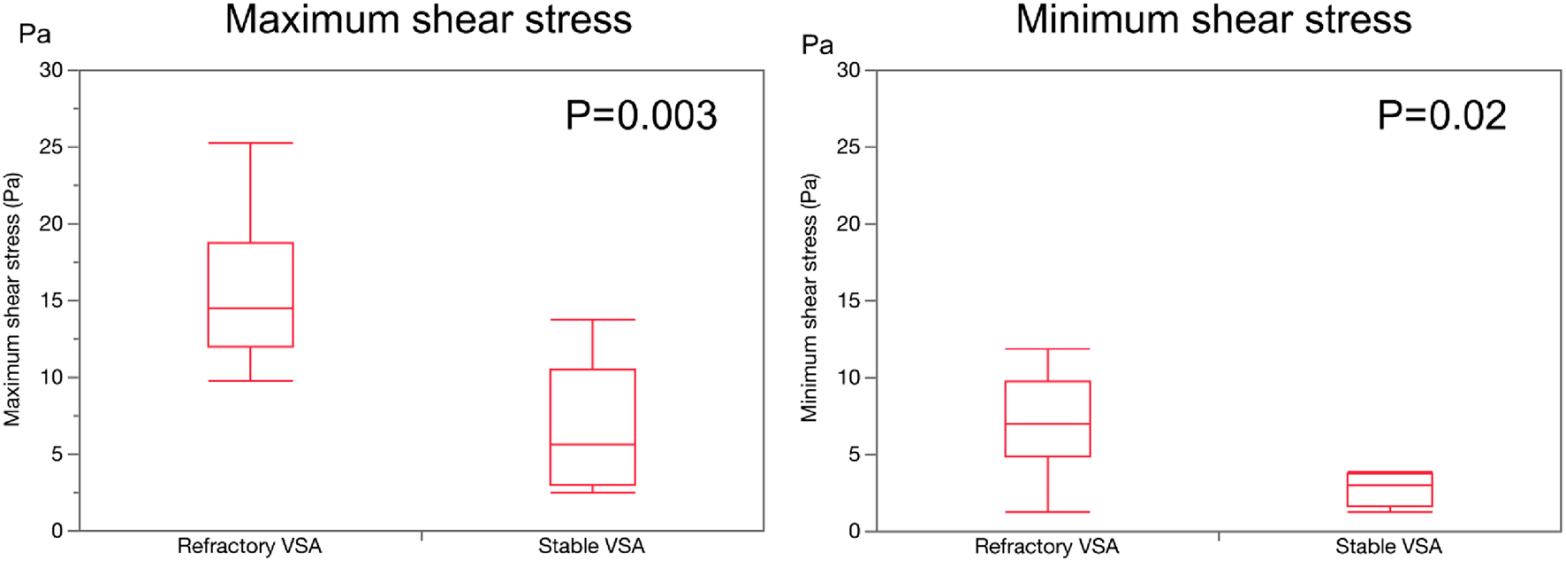
Maximum and minimum shear stress. The left panel presents maximum shear stress and the right panel shows minimum shear stress of both groups. The maximum shear stress in the refractory VSA group was higher than that in the stable VSA group (refractory VSA 14.5 [12.1–18.8] Pa vs stable VSA 5.6 [3.0–10.5] Pa, p = 0.003). The minimum shear stress was also higher in the refractory VSA group compared to that in the stable VSA group (refractory VSA 7.0 [4.9–9.7] Pa vs stable VSA 3.0 [1.6–3.8] Pa, p = 0.02).

Figure 3 shows the maximum and minimum shear stress of the two groups. The maximum shear stress in the refractory VSA group was higher than that in the stable VSA group (refractory VSA group 14.5 [12.1–18.8] Pa vs stable VSA group 5.6 [3.0–10.5] Pa, p = 0.003). The minimum shear stress was also higher in the refractory VSA group compared to that in the stable VSA group (refractory VSA group 7.0 [4.9–9.7] Pa vs stable VSA group 3.0 [1.6–3.8] Pa, p = 0.02).

**Figure 4:**
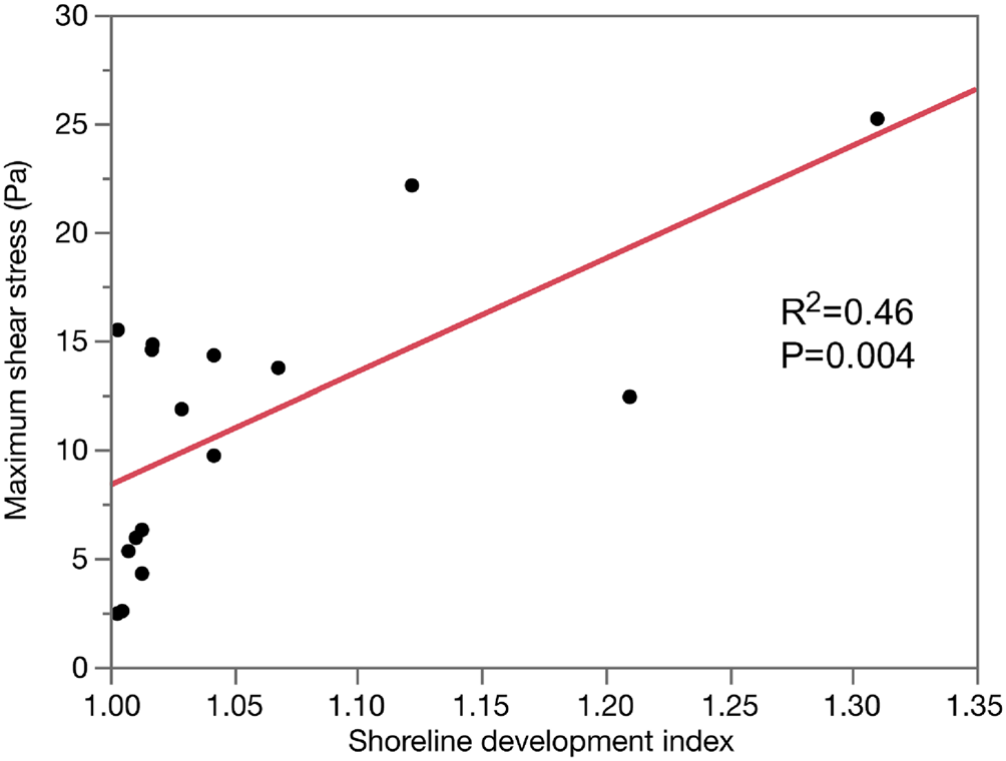
Shoreline development index and maximum shear stress. Shear stress is positively correlated with the shoreline development index (R^2^ = 0.46, P = 0.004). Complexity of lumen circumference increases the shear stress in VSA.

Figure 4 shows the relationship between the shoreline development index and shear stress. The shoreline development index positively correlates with shear stress (R^2^ = 0.46, P = 0.004).

## Discussion

This study revealed a thicker media of the coronary artery, more complex circumference of the coronary lumens, and higher maximum shear stress in patients with VSA who had refractory symptoms compared to those without refractory symptoms.

The underlying mechanisms for the development of VSA are not fully understood and several etiologies have been proposed, including endothelial dysfunction^11^, oxidative stress^12^, inflammation^13^, and genetic disorders^14^. Among them, endothelial dysfunctional involving NO synthase has been thought to contribute to the development of VSA^1,4,11^. However, several reports question this hypothesis, since endothelial dysfunction is not always associated with VSA^15,16^. There has been a prolonged discussion on the pathophysiology of VSA and the associated factors are yet to be classified into etiological or predisposing factors. To date, hyper-reactivity of the vascular smooth muscle in the medial layer is believed to be the primary pathophysiological mechanism responsible for epicardial coronary artery spasm^17^. Shimokawa et al*.* developed a porcine model of VSA and showed that excessive-reactivity of the media caused by upregulated Rho-kinase activity is an essential factor for the development of VSA^17,18^. The Rho-kinase activity in the peripheral leukocytes independently predicts the severity of VSA^19^. These findings are consistent with our observation that VSA with refractory symptoms is associated with a thicker medial layer. The intimal bump induced by excessive-medial constriction is one of the key morphological features of VSA lesions^9^. In this study, despite we continued with the use of vasodilators, the intimal bump occurred numerically more often in the refractory group, but the difference was not statistically significant, probably due to the small sample size. Furthermore, the circumferential shoreline of the coronary lumen was more complex in the refractory VSA group compared to that in the stable VSA group. Uchida et al*.* reported that the radial rearrangement of the vascular smooth muscle cells in the media, by their own contraction result in medial thickening and folding of the internal elastic lamina, which creates the complex luminal narrowing^20^. The unique morphology of the coronary lumen in the VSA model is very similar to that of our OCT images from VSA patients. These findings could indicate a link between excessive-medial constriction, intimal bumps, and circumferential lumen complexity in VSA.

In the normal vascular homeostasis, high shear stress reduces the vascular tone and relaxes the vascular lumen via NO synthesis^7,8^. In contrast, in this study, we found that the lumen shoreline complexity correlated with shear stress. We speculate that NO synthesis in patients of VSA with refractory symptoms is already up-regulated and exhausts its reserve due to unexpected high shear stress caused by medial over-constriction and lumen complexity. Hence, it loses its ability to respond to faint vasoconstrictive stimuli, such as the autonomic nervous system^21^, smoking^22^, exposure to cold^23^, hyperventilation and alkalosis^24^, thereby triggering frequent VSA symptoms. Previtali et al*.* showed that sensitivity of the hyperventilation test, which has low sensitivity for the detection of VSA under normal circumstances, was similar to that of the pharmacological provocation test in a patient who had more than 1 episode of VSA per day^25^, which supports our hypothesis. In addition, since the main pathophysiology of VSA is reversible epicardial coronary artery stenosis, blood rheology in the culprit site can change dramatically, spatially, and temporally. Rapid changes in local shear stress induce stabilized aggregation of the discoid platelets, resulting in release of granules with vasoconstrictors^26,27^. A high shear stress induced platelet aggregation might contribute to aggravating the symptoms of VSA. Further studies are necessary to confirm our hypothesis.

Various vasodilators, including calcium channel blockers, are used in the treatment of VSA. A large registry in Japan has shown good prognosis of VSA under treatment with vasodilators^3^. However, a Korean group reported that more intensive clinical attention is necessary for VSA patients with frequent angina under medical treatment^6^. Hence, it is important to discriminate between VSA patients with stabilized medial constriction by medical therapy and those without. The SDI is objective and very convenient for OCT because the current commercially available OCT systems contain an automatic lumen edge detection system that can instantly calculate the SDI. Further studies should be conducted to validate this possibility.

Our study has several limitations. First, this was a single center, small cohort, observational study, and we did not assess the NO metabolite. Therefore, the relationship between high shear stress and NO is still unknown. No flow parameters were -assessed in this study and in the process of shear stress estimation. We did not mention curvatures of the coronary artery in the estimation of shear stress. Kumar et al. estimated shear stress using fractional flow reserve and angiograms to predict future events in patients with obstructive coronary artery disease^28^. Since their estimation of shear stress considered the vascular curve, it appears very superior with more precise estimation. Under the presence of coronary stenosis, shear stress is significantly impacted by the severity of stenosis. Their method could be apt for the objective of their study. However, patients with VSA do not show severe organic stenosis. Therefore, we used our method that preferred the details of lumen contour derived by OCT. Siosos et al. estimated shear stress from the reconstructed 3D anatomy of the coronary artery by angiography and intravascular ultrasound, in non-obstructive coronary artery disease^29^. Since our study required details of the coronary artery lumen contour, we used OCT rather than intravascular ultrasound. OCT acquires multiple images spirally and reconstructs the cross-sectional image. Therefore, it is impossible to precisely superimpose the OCT image on to the angiography image. Finally, patients with VSA do not always mention all their symptoms.

In conclusion, increased medial thickness of the coronary artery provokes lumen complexity and increases shear stress, which might in turn cause refractory symptoms in patients with VSA. Shear stress estimated from the OCT images and the shoreline development index could serve as a marker for irritability of the medial layer of coronary arteries and help in evaluation of the drug efficacy in the future.
